# Evaluation of Chondroprotective Activity of Channa striatus in Rabbit Osteoarthritis Model

**DOI:** 10.1155/2019/6979585

**Published:** 2019-07-03

**Authors:** Azidah Abdul Kadir, Arifah Abdul Kadir, Roslida Abd Hamid, Abdul Manan Mat Jais, Julia Omar, Abdul Nawfar Sadagatullah, Salziyan Badrin, Thin Thin Win, K. N. S. Sirajudeen, Annas Salleh

**Affiliations:** ^1^Faculty of Veterinary Medicine, Universiti Putra Malaysia, Universiti Putra Malaysia, Serdang, 43400 Selangor, Malaysia; ^2^Department of Family Medicine, School of Medical Sciences, USM Health Campus, 16150 Kubang Kerian, Kelantan, Malaysia; ^3^Department of Veterinary Preclinical Sciences, Faculty of Veterinary Medicine, Universiti Putra Malaysia, 43400 Selangor, Malaysia; ^4^Department of Biomedical Science, Faculty of Medicine and Health Sciences, Universiti Putra Malaysia, 43400 Selangor, Malaysia; ^5^Abmanan Biomedical Sdn Bhd (ABSB), A-G-1, Univ 360 Place, Jalan Raya 2, Taman Serdang Raya, 43300 Seri Serdang, Selangor, Malaysia; ^6^Department of Chemical Pathology, School of Medical Sciences, USM Health Campus, 16150 Kubang Kerian, Kelantan, Malaysia; ^7^Department of Orthopaedic, School of Medical Sciences, USM Health Campus, 16150 Kubang Kerian, Kelantan, Malaysia; ^8^Medical Faculty, International Medical University, No. 126, Jalan Jalil Perkasa 19, Bukit Jalil, 57000 Kuala Lumpur, Malaysia

## Abstract

**Objectives:**

The objective of the study is to evaluate the chondroprotective activity of* Channa striatus* (Channa) and glucosamine sulphate (glucosamine) on histomorphometric examinations, serum biomarker, and inflammatory mediators in experimental osteoarthritis (OA) rabbit model.

**Design:**

Anterior cruciate ligament transection (ACLT) was performed to induce OA in thirty-three male New Zealand white rabbits and were randomly divided into three groups: Channa, glucosamine, and control group. The control group received drinking water and the Channa and glucosamine groups were orally administered with 51.4 mg/kg of Channa extract and 77.5 mg/kg of glucosamine sulphate in drinking water, respectively, for eight weeks and then sacrificed. The articular cartilage was evaluated macroscopically and histologically using semiquantitative and quantitative methods. Serum cartilage oligomeric matric protein (COMP), cyclooxygenase 2 (COX-2) enzyme, and prostaglandin E_2_ (PGE_2_) were also determined.

**Results:**

Macroscopic analysis revealed that Channa group have a significantly lower severity grade of total macroscopic score compared to the control (p < 0.001) and glucosamine (p < 0.05) groups. Semiquantitative histology scoring showed that both Channa and glucosamine groups had lower severity grading of total histology score compared to the control group (p < 0.001). In comparison with the control, Channa group had lower histopathological changes in three compartments of the joint compared to glucosamine group which had lower histological scoring in two compartments only. The cartilage thickness, area, and roughness of both Channa (p < 0.05) and glucosamine (p < 0.05) groups were superior compared to the control group. However, the Channa group demonstrated significantly less cartilage roughness compared to the glucosamine group (p < 0.05). Serum COMP levels were lower in both Channa (p < 0.05) and glucosamine (p < 0.05) groups compared to the control group.

**Conclusion:**

Both oral administration of Channa extract and glucosamine exhibited chondroprotective action on an ACLT OA-induced rabbit model. However, Channa was superior to glucosamine in maintaining the structure of the cartilage.

## 1. Background

The prevalence of knee osteoarthritis (OA) is expected to increase globally due to the rising increments of an aging population and obesity [1]. The management of knee OA is challenging since currently there is no pharmacological agent recognised as being a structure (disease) modifying agent that is able to retard the disease process [2]. Pharmacological agents are used to control the disease symptoms; they are traditional and cyclooxygenase-2 selective nonsteroidal anti-inflammatory drugs (NSAIDs) which carry the risk of gastrointestinal and cardiovascular side effects [2, 3]. Therefore, there is an increasing research interest in identifying a pharmacological agent that is able to prevent or reduce OA progression [2].

*Channa striatus* (Channa), a snakehead freshwater fish belonging to the Channidae family, is one of the well-known traditional medicines used for wound healing in South East Asia countries, especially Malaysia. Its use in treating knee osteoarthritis [4] has been explored due to its anti-inflammatory [5–7], analgesic [6, 8], and wound healing properties [9, 10].* In vivo* studies using a rabbit OA model showed that there was a reduction of the soft tissue swelling of the joint and it also reduced the density of the protein gene product (PGP) 9.5-immunoreactive nerve fibres in the synovial membrane of the Channa-treated group compared with the control [11]. In another animal study using rats induced with OA, the levels of serum prostaglandin E_2_ (PGE_2_) were significantly reduced in the Channa-treated animals compared to the rats treated with celecoxib (a group of COX-2 inhibitors) [12].

The Channa extract was produced through Pressurised In-Water Extraction and a proximate analysis was used to standardise the extract that contained the protein up to 78.32 + 0.23%, fat 2.08 + 0.08%, and Vitamin A at 0.27 + 0.01%. The suspected bioactive compound of the Channa extract was a macromolecule, a short-chain peptide N-arachidonylglycine. The studies conducted found that the Channa extract was rich with 17 amino acids including glycine, glutamic acids, arginine, and aspartic acid, which is nonessential [13, 14]. Some of the abundant fatty acids in the CS extracts were C16:0 (palmitic acid), C18:0 stearic acid), C18:1 (oleic acid), C20:4 (arachidonic acid), and docosahexaenoic acid (DHA) (C22:6).

Cartilage oligomeric matrix protein (COMP) is a biomarker of cartilage degradation and it has been used for monitoring OA progression and determining OA severity [15–17]. Serum COMP has also been shown to correlate closely with knee OA severity and with the number of joints affected [18, 19]. COX-2 is an enzyme that leads to the formation of prostaglandins including prostaglandin E_2_ (PGE_2_) and thromboxane [20]. Evidence has shown that PGE_2_ and COX-2 synthesis are upregulated in OA [20]. A number of studies revealed that PGE_2_ has been involved in the modulation of the tissue destruction observed as occurring in OA, such as proteinase activation, matrix protein synthesis, cell proliferation/apoptosis, and the sensitisation of nociceptors [21, 22].

Channa extract could be an alternative therapy for knee OA patients which can reduce the use of NSAIDs and its complications. The chondroprotective potential of Channa has been reported in recent* in vivo* studies (Al-Saffar et al., 2011a; Michelle et al., 2004). No comparison study has been conducted yet with glucosamine, which has been widely taken to reduce the pain and stiffness that is due to OA. The aim of the study is to evaluate the chondroprotective effect of oral Channa extract versus glucosamine by using macroscopic, semiquantitative, and quantitative histological grading and to evaluate the serum cartilage degradation biomarker, COMP. This is as well as the inflammatory markers such as COX-2 and PGE_2_ in ACLT OA-induced rabbit model.

## 2. Methods

### 2.1. Ethical Statement

The study protocols were approved by the Animal Ethics Committee of Universiti Sains Malaysia (USM) (USM/animal ethics approval/2015/(97) 686). All animal handling and experimentation were performed in accordance with the National Advisory Committee on Laboratory Animal Research (NACLAR) Guidelines on the care and use of animals for research [23].

### 2.2. Experimental Animals

Thirty-three adult male New Zealand white rabbits that were 7-8 months old, weighing between 2.0 and 3.0 kg, were used as the experimental animals in this study. The rabbits were provided by a local vendor registered with Universiti Sains Malaysia (USM) and they were given one week to acclimatise to the housing facility. The rabbits were kept under a 12-hour ligh-dark cycle. They were housed in individual stainless steel cages (450 X 600 X 450 mm) and permitted free access to food pellets and water. The rabbits' housing, daily monitoring, and experimental procedures were conducted in the Animal Research and Service Centre (ARASC) of Health Campus USM.

### 2.3. Animal Preparation

The rabbits underwent unilateral ACLT under general anaesthesia. The rabbits were anaesthetised preoperatively with an intramuscular administration of ketamine 35 mg/kg and xylazine 5mg/kg. Anaesthesia for the surgical procedure was maintained with a mixture of isoflurane in oxygen. The surgery was carried out in the standard manner. A medial arthrotomy was performed on the right femoropatellar joint to permit the transaction of the anterior cruciate ligament. Tramadol hydrochloride (UNICHEM-India) 2 mg/kg once daily was given for 3 days following the surgery. Antibiotics (sulphamethoxazole 5 mg and trimethoprim (TMP) 1 mg 24%) were given subcutaneously twice a day preoperatively and for 2 days postoperatively. Postoperatively, all animals were permitted free cage activity.

### 2.4. Experimental Procedure

The rabbits were randomly divided into 3 groups using block randomisation in a block of six three weeks after the ACLT procedure:* Channa* (n = 11), glucosamine (n = 11), and the control (n = 11). The rabbits in the Channa group were administered with 51.4 mg/kg of spray dried Channa extract, while the glucosamine group received 77.5 mg/kg of glucosamine sulphate and both were dissolved in drinking water. The control group received only drinking water. The dose for Channa and glucosamine was based on the human study and the conversion from human to rabbit dose was done according to Jang-Woo Shin* et al.* (2010) [24]. The dose for Channa was based on 1000mg for a 70 kg human per day [25] and the dose for glucosamine was based on 15000mg for a 70 kg human per day [26]. The investigational product was orally administered to the rabbits via a syringe for a period of 8 weeks starting 3 weeks after the surgery before they were euthanised by an intravenous Phenobarbitone overdose. The blood collection was conducted via venipuncture for COMP, COX-2, and PGE2 before they were sacrificed. The serum was separated from the collected blood and stored at -80° Celsius until further use. The spray-dried Channa extract powder was supplied by Prof. Abdul Manan Mat Jais from Universiti Putra Malaysia, Malaysia.

### 2.5. Macroscopic Cartilage Assessment

The gross morphological assessment of the knee joints was conducted according to the Indian ink staining method [27]. The macroscopic method of Indian ink staining is a method used for highlighting areas of cartilage degeneration and providing a broad morphological view of the cartilage degeneration [28]. The femoral-tibial joint compartments were divided into four groups: medial femur (MF), lateral femur (LF), medial tibia (MT), and lateral tibia (LT). The grading used was as follows: for grade 1 (intact surface), the surface appears to be normal and does not retain any ink. For grade 2 (minimal fibrillation), the minimal focal uptake of India ink indicates mild surface irregularity. For grade 3 (overt fibrillation), there is evidence of large focal dark patches of ink uptake showing overt fibrillation. For grade 4 (erosion), the loss of cartilage is evident with the exposure of the bone. The gross morphological assessment of the knee joints was performed in a blinded fashion by a pathologist and an orthopaedic surgeon.

### 2.6. Semiquantitative Histology Assessment

Histologic evaluation was performed on the sagittal sections of cartilage from the lesions on the femoral condyles and tibial plateaus. The tissue blocks were fixed in 10% neutral buffered formalin and decalcified with 5% nitric acid for 72–120 hours. When decalcification was completed, the femoral and tibial condyles were divided sagittally into two equal parts and embedded in paraffin. Four *μ*m sections were cut at a standard site centrally and stained with hematoxylin-eosin staining.

The semiquantitative histology assessment was assessed and compared according to the modified Osteoarthritis Research Society International (OARSI) scoring system [27, 29]. This scale evaluates the histopathological changes in each animal based on structure (scale 0–11), chondrocyte density (scale 0–4), and cluster formation (scale 0–3) (Table 1). The final score corresponds to the score of the most severe lesions. All of the assessment was performed by a pathologist.

### 2.7. Quantitative Histology Assessment

The medial femoral condyles were selected for use in the quantitative histology assessment. This is in accordance with the previous studies that showed that medial femoral condyles had the most advanced changes and that they were used for the associated quantitative histology assessment [30, 31]. The quantitative histologic assessment method used in this study was based on a study by Amiel* et al.* (2003) [32] and Shimizu* et al.* (1998) [30]. All assessments were done by a single researcher. The histological sections were visualised using a high-resolution image analyser (Olympus BX41, Olympus Australia, PTY, LTD) that was analysed with a computer image analysis software (Olympus Soft Imaging Solutions, Olympus Australia, PTY, Ltd.). The customised image analysis software measured the following geometric parameters: cartilage thickness, cartilage area, and the surface roughness of the cartilage.

**Figure 1:**
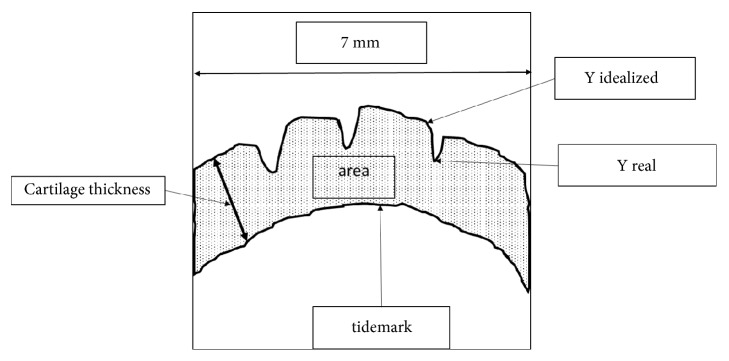
Schematic representation of typical histology specimen used for quantitative histology assessment.

The geometric parameters of the cartilage specimens were measured using a 7mm area of the medial femoral condyle at 40X magnification (Figure 1). A 7 mm weight bearing section of the femoral condyle was defined through the area of the greatest damage to the medial femoral condyle. The distance scale was calibrated before analysis by the means of a standard precision where a 1 mm scaled ruler was placed under the microscope and its length in *μ*m was measured in the computer image analysis software. The thickness of the cartilage from the surface to the tidemark was calculated from the mean of 20 measurements made perpendicularly to the surface of each section at equally spaced points (Figure 1). The area of the cartilage present (the 7 mm greatest damage of femoral condyle) was calculated (Figure 1). The thickness and area of cartilage were computed using the coordinates of the articular cartilage and the tide mark.

Calculation of cartilage roughness is based on deviations from an idealized smooth surface which is derived from shape parameters of normal cartilage outside the region of degeneration. This parameter is expressed as root mean square (RMS) surface roughness calculated for the following equation [30, 32]:

(1) RMS  surface  roughness=1N+∑i=1NY  idealizedi−Y  reali21/2.

N is the number of digitized points.

Y idealized_i_ is the theoretical coordinate of the ideal smooth surface of articular cartilage, determined from the coordinates of surrounding normal cartilage surface.

Y real_i_ is the actual coordinate of articular cartilage surface.

Since surface roughness is dependent on surface thickness [32], therefore, calculation of surface roughness normalized to cartilage thickness was made:

(2) Normalized  cartilage  roughness=RMS  surface  roughnessCartilage  thickness.

### 2.8. Estimation of Cartilage Degradation Biomarker and Inflammatory Markers

Serums COMP, COX-2, and PGE_2_ were measured using a double-antibody sandwich enzyme-linked immunosorbent (ELISA) one-step process (Qayee-Bio Technology Co., Ltd. Shanghai, China). The detection range of the kits was 1.56 – 100ng/ml.

### 2.9. Statistical Analysis

The sample size was calculated using PS: Power and Sample Size Calculations software for comparing two means (Type I error of 5% and Type II error of 10%). The sample size was determined based on the assumption that the largest difference would be observed between CS and the placebo macroscopic score. It was determined that a sample size of 10 in each group was needed to detect a difference of 0.32 with a standard deviation of 0.5 [33]. Eleven rabbits were enrolled in each group, to allow for a 10% dropout rate.

Analyses were performed using SPSS for Windows version 22.0 (SPSS Inc. Chicago, Illinois, USA). The data distributions for each parameter were initially determined by normality tests (Shapiro-Wilk test, histogram, and Box-plot). One-way analysis of variance (ANOVA) with post hoc Tukey's test or Dunnet's C test was used to analyse the histomorphometric assessment, serum COMP, COX 2, and PGE_2_. Post hoc Tukey's test was used when homogeneity of variances was met (Levene's test p value > 0.05) and post hoc Dunnet's C test was used when homogeneity of variances was not met (Levene's test p value < 0.05). Kruskall Wallis Test followed by repeated Mann Whitney test for each pair with adjusted p values was used to analyse the gross morphologic assessment since the variable exhibited nonnormal distribution. A p value < 0.05 was regarded as statistically significant difference for all tests.

## 3. Results

### 3.1. Clinical Observation

All the animals recovered uneventfully from the OA induction and none of the animals were lost in the study. No adverse event was observed.

### 3.2. Macroscopic Assessment

**Figure 2:**
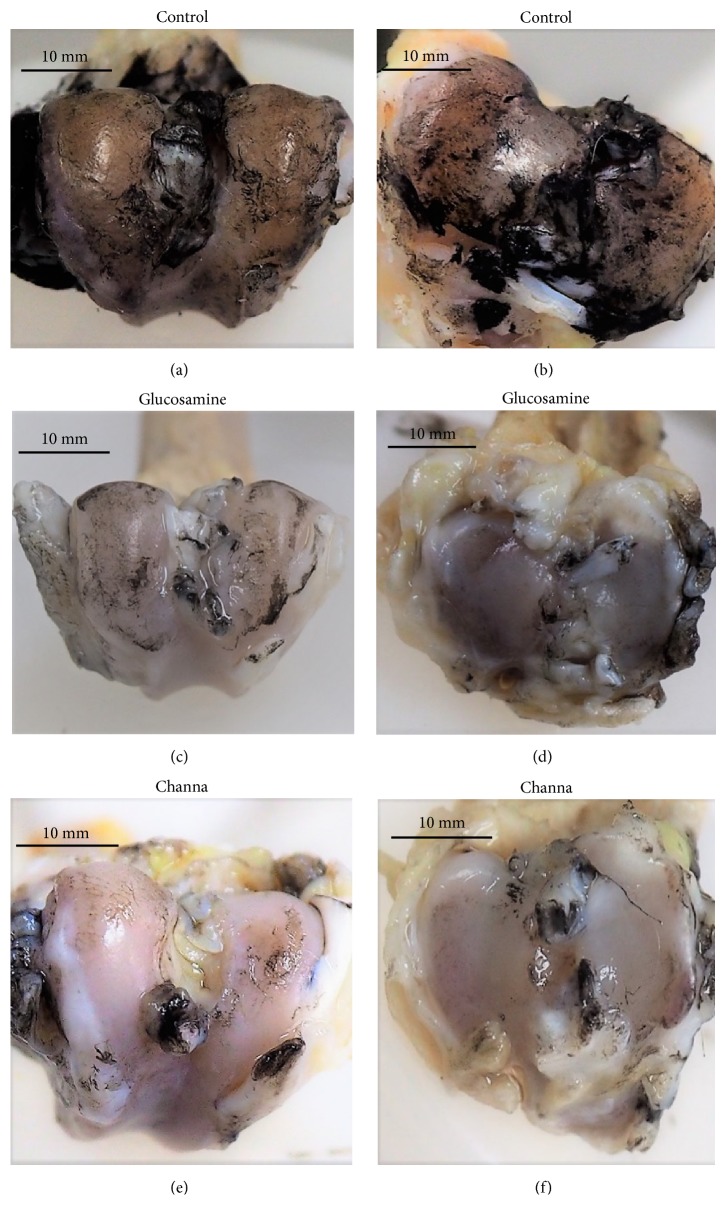
Macroscopic representative of the treatment groups. The control group ((a) and (b)) had more intense black patches on the articular surfaces indicating area of fissures or fibrillation compared to glucosamine ((c) and (d)) and Channa ((e) and (f)).

Figure 2 showed the representative of the macroscopic changes of articular cartilage according to the experiment groups. The images showed that the control group had higher severity grading compared to Channa and glucosamine groups. As seen in Table 2, the control groups exhibited severe gross morphological assessment compared to other treatment groups. The Channa group (median 4.00 IQR 2.00) have a significantly lower severity grade of total macroscopic score compared with the control (10.00 IQR 2.00) (p < 0.05) and glucosamine (9.00 IQR 3.00) (p < 0.05) groups. The total macroscopic score analysis of the joint demonstrated that there was no significant difference between glucosamine and control groups.

In the Channa-treated group, there was marked reduction of macroscopic score compared to the control group in all joint compartments (p < 0.05). In comparison, the glucosamine group have a significantly lower severity grade of macroscopic score compared to the control group in the medial femur condyle (p < 0.05). There was also a significant difference between Channa and glucosamine group in lateral femoral condyle (p < 0.05) and lateral tibial plateau (p < 0.05).

### 3.3. Semiquantitative Histological Grading

**Figure 3:**
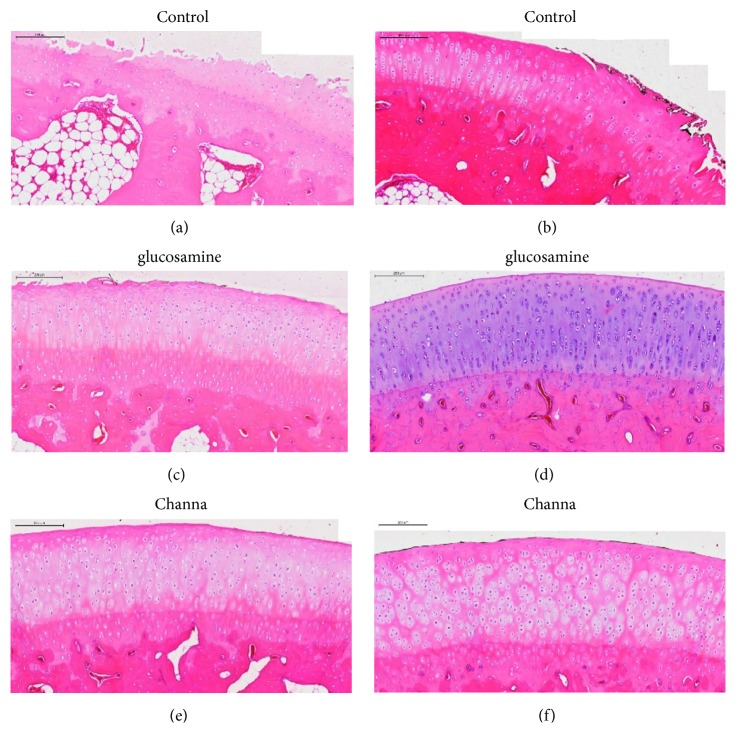
Sample histological sections of the treatment groups (magnification 10X). The control group ((a) and (b)) demonstrated higher severity grading of the structure component evidence by presence of erosion, fissures, and more chondrocyte loss compared to glucosamine ((c) and (d)) and Channa ((e) and (f)).

**Figure 4:**
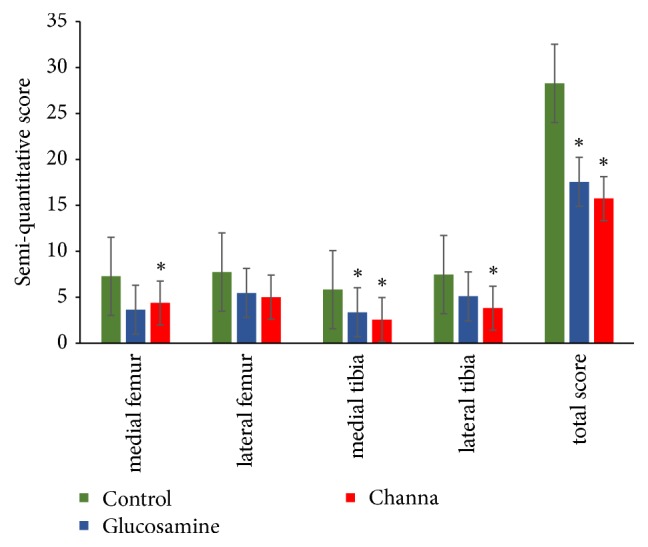
Scores for histology semiquantitative grading in Channa, glucosamine, and control groups. Data are presented as mean ± SEM (n = 11 per group). Significant differences determined by one-Way ANOVA followed by Tukey's post hoc test. ^*∗*^ p<0.05 compared with the control group.

Histological assessment based on OARSI scoring system showed that animals treated with Channa and glucosamine had a trend towards reduced severity of cartilage lesions compared to control group (Figures 3 and 4). Overall, the control group (mean 28.27 ± SEM 1.77) have higher severity grading compared to glucosamine (mean 17.55 ± 1.93) (p<0.05) and Channa groups (mean 15.73 ± 1.56) (p<0.05) (one-way ANOVA). There was no statistical difference found between Channa and glucosamine groups (p= 0.845).

Detailed analysis indicated that CS significantly had lower degenerative changes compared to the control groups in three compartments of the joint: medial femur (Channa mean 4.36 ± 0.57, control 7.27 ± 0.68) (p < 0.05), medial tibia plateau (Channa 2.55 ± 0.31, control 5.82 ± 0.77) (p < 0.001), and lateral tibia plateau (Channa 3.82 ± 0.82, control 7.45 ± 0.79) (p < 0.05). In comparison, glucosamine (3.64 ± 1.34) had significantly lower severity grading compared to the control group (7.27 ± 0.77) in medial femur and medial tibia plateau (p < 0.05) (one-way ANOVA).

### 3.4. Quantitative Histology Grading

Histomorphometrically, control group (mean 155.73 ± SEM 19.50 *μ*m) had significantly lower cartilage thickness compared to the Channa (242.82 ± 12.79 *μ*m) (p < 0.05) and glucosamine (211.73 ± 10.60 *μ*m) (p < 0.05) groups. Both Channa (97,722.27 ± 56,189.26 *μ*m^2^) (p < 0.001) and glucosamine (79,368.91 ± 17,743.20 *μ*m^2^) (p < 0.05) groups also demonstrated higher cartilage surface area than the control group (57,895.82 ± 64,355.63 *μ*m^2^).

**Figure 5:**
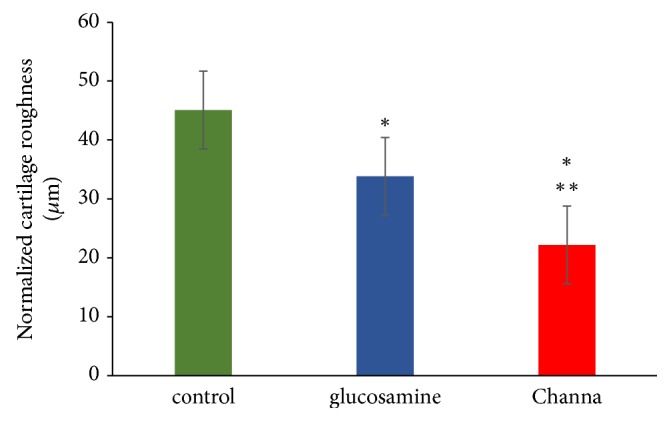
Normalized cartilage roughness (RMS roughness/cartilage thickness) of the medial femoral condyles (*μ*m) Note: results represent mean ± SEM. Significant differences determined by one-way ANOVA followed by Tukey's post hoc test. ^*∗*^ p < 0.05 compared with control group, ^*∗∗*^ p < 0.05 compared with glucosamine group.

The control group (45.10 ± 4.17*μ*m) also demonstrated higher normalized cartilage roughness compared to both Channa (22.18 ± 2.35 *μ*m) and glucosamine (33.82 ± 2.17 *μ*m) (p < 0.05) groups (Figure 5). There was no significant difference between Channa and glucosamine groups in terms of cartilage thickness and area. However, it was noted that the Channa group had significantly lower readings of normalized cartilage roughness than the glucosamine group (p < 0.05).

### 3.5. Cartilage Degradation Biomarker and Inflammatory Markers

**Figure 6:**
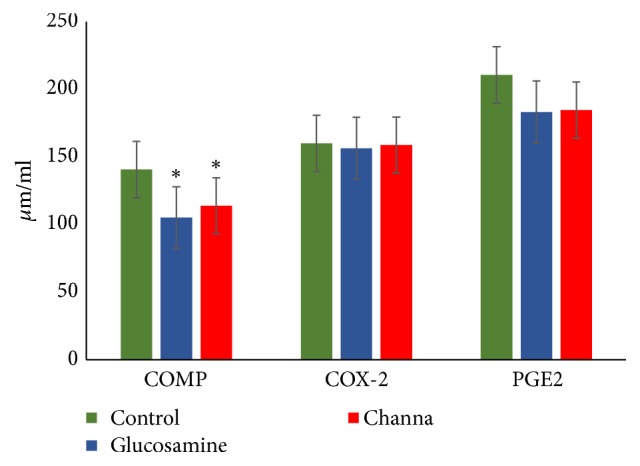
Serum COMP, COX-2, and PGE_2_ among Channa, glucosamine, and control groups. Note: results represent mean ± SEM. Significant differences determined by one-way ANOVA followed by Dunnet's post hoc test. *∗* p < 0.05 compared with control group.

Serum level of COMP, a biomarker of cartilage degradation, was significantly high in the control group compared to Channa and glucosamine groups (p < 0.05) (Figure 6). There were no significant differences between all the treatment groups in serum COX-2 and PGE_2_ levels.

## 4. Discussion

This is the first* in vivo* study that compares Channa and glucosamine in knee OA. The findings of the semiquantitative histology assessment were further supported by the quantitative histomorphometric assessment conducted using parameters such as surface roughness, cartilage area, and thickness. The semiquantitative histological assessment of articular cartilage using scoring systems such as the Mankin grading system was considered to be the gold standard for the evaluation of the severity of osteoarthritis in the animal models [27]. However, this is a subjective scoring system; thus, the histomorphometry measures employed a computer-based image analysis system to objectively assess the histochemical characteristics of the articular cartilage. In our study, the use of the histology quantitative scoring method complements the semiquantitative scoring method, which is more subjective [34].

Glucosamine was chosen as a positive control in this study since it is a popular oral supplement globally used by knee OA patients [35, 36]. The animal models showed that it improved the cartilage lesions compared with the controls [33, 37, 38] and that it also involves anti-inflammatory activity [36, 39]. Clinical studies on the effect of glucosamine for OA have yielded mixed results. Most of the meta-analysis and reviews showed that glucosamine did have some effect when it came to relieving the symptoms, with the structural effect of joint space narrowing [34, 36].

The gross morphology and histomorphometric findings indicated that both Channa and glucosamine showed a better pattern of tissue organisation, with less fibrillation and erosion, cartilage thickness, and chondrocyte organisation compared to the control group. There was less chondrocyte apoptosis. This is evidenced by the histological analyses that showed that the loss of chondrocytes was less in the Channa and glucosamine treated-groups. The cartilage thickness, area, and roughness provide further evidence of the chondroprotective effect of Channa and glucosamine. However, Channa showed less cartilage roughness compared to glucosamine; thus, this showed that Channa had a better pattern of tissue organisation compared to glucosamine. The results support the use of Channa as a disease/structure modifying drug used to reduce the progression of articular cartilage degeneration in OA. The findings of this study were similar to the animal study conducted by Al-Saffar* et al. *(2011) using monosodium iodoacetate, which was used to induce arthritis in rats. They compared the oral CS extract, Celecoxib, and the control (normal saline). However, compared to the study by Saffar* et al. *(2011), this study used a wide range of morphological grading and histological assessment including quantitative histological grading and biomarkers for cartilage degeneration.

In this study, we found that the Channa extract prevents fibrillation and surface irregularities, thus reducing the friction of the joint. Cartilage roughness indicates degeneration and it is also part of the normal circumstances of repair [28]. This finding may indicate that Channa acts through a wound healing mechanism [9, 40]. Orally administered extract of Channa has been shown to induce healing in experimentally induced gastric ulcers in Wistar rats [41]. A clinical trial conducted among post-Caesarean women also demonstrated that there was a significant better wound cosmetic appearance and uterus involution in the women treated with oral Channa compared to the placebo group [42, 43]. The wound healing properties of CS are contributed to by the presence of fatty acid and amino acids, especially glycine and arachidonic acid [44]. Channa extract is believed to promote wound healing by initiating collagen synthesis and reepithelialisation in the damaged tissues [44].

The serum levels of COMP, a biomarker of cartilage degeneration, were significantly high in the control group compared to both Channa and the glucosamine group. Higher levels of serum COMP in the control group indicates that more cartilage degradation has occured [15]. Serum COMP has been shown to predict variations in joint remodelling, cartilage loss, and the depletion of the extracellular matrix [15]. The reduction of serum COMP in both the Channa and glucosamine groups supports the macroscopic and histomorphometric findings.

The results of the inflammatory markers were not conclusive. No difference was found in terms of the PGE_2_ and COX-2 serums between all treatment groups. The findings of serum PGE_2_ in our study contradict the findings by Al Saffar* et al.*, who noted that the rats administered with oral Channa had levels of PGE_2_ that were reduced significantly, comparable to the group treated with celecoxib (COX-2 inhibitor) [12]. Discrepancies between the results of our study and those of Al Saffar* et al. *[12] may be explained by a few factors. These include the differences in the OA model used, the biological variations due to the different species of animal, and the dose used in Al Saffar* et al.*'s study [12], which was 40 times more compared to our study.

We postulate that Channa improves the anabolic activity in the extracellular matrix component through its action of increasing the synthesis of glycosaminoglycan (GAG) and hyaluronic acid [9]. The improvement of the matrix component in OA by the Channa extract has also been shown by the improvement of the Safranin O fast green staining in terms of the histological assessment of the articular cartilages in an animal study [12]. The incremental increase in GAG will increase the proteoglycans aggregates and strengthen the articular cartilage [4].

The exact mechanism of action of the Channa extract on osteoarthritis is still largely unknown. It is possible that Channa works through anti-inflammatory [12, 45], wound healing [9, 10, 42], and analgesic [6, 8] properties. The possible presence of various compounds with multiple modes of action provides the challenge of elucidating the exact mechanism of the actions. Recently, a bioactive fraction has been isolated from the fish fillet known as striatin (DLBS0333) [46]. This protein fraction has been shown to contain four major bioactive proteins including amino acids that are essential to enhancing wound healing such as linoleic acid, palmitic acid, and glycine [46]. The* in vivo* study demonstrated that this compound enhanced fibroblast proliferation and enhanced wound healing in a wound-induced rat model [46].

Glycine was one of the amino acids detected in the CS extract [46]. It has been shown that glycine helps in the remodelling of collagen via the synthesis of inter- and intramolecular protein linking [9]. It also acts synergistically with other essential amino acids like proline, alanine, arginine, isoleucine, phenylalanine, and serine to form a polypeptide that promotes tissue repairing and healing process [47]. The CS extract also had a high amount of arginine and arginine supplementation has been observed to enhance the amount of collagen deposited into a standardised wound [48].

The histological analysis in this study was done using hematoxylin-eosin staining only and we did not measure the GAG content of the cartilage. A future study to assess the efficacy of the combination of Channa and glucosamine in the treatment of knee osteoarthritis (OA) is recommended.

## 5. Conclusions

We demonstrated that oral administration of Channa extract exhibits chondroprotective action on an ACLT OA-induced model. Channa was also superior to glucosamine in maintaining the structure of the cartilage. These results indicate that the long-term structure-modifying effects of Channa should be further evaluated in patients with OA of the knee.

## Figures and Tables

**(a):** 

0	normal
1	Surface irregularities
2	Fissures in < 50% surface
3	Fissures in ≥ 50% surface
4	Erosion 1/3 hyaline cartilage < 50% surface
5	Erosion 1/3 hyaline cartilage ≥ 50% surface
6	Erosion 2/3 hyaline cartilage < 50% surface
7	Erosion 2/3 hyaline cartilage ≥ 50% surface
8	Full depth erosion 2/3 hyaline cartilage < 50% surface
9	Full depth erosion 2/3 hyaline cartilage ≥ 50% surface
10	Full depth erosion hyaline cartilage and calcified cartilage to the subchondral bone < 50%
11	Full depth erosion hyaline cartilage and calcified cartilage to the subchondral bone ≥ 50%

**Table:** (b) Chondrocyte density

0	No decrease in cells
1	Focal decrease in cells
2	Multifocal decrease in cells
3	Multifocal confluent decrease in cells
4	Diffuse decrease in cells

**Table:** (c) Cluster formation

0	normal
1	< 4 clusters
2	≥ 4 but < 8 clusters
3	≥ 8 clusters

**Table 2:** Macroscopic grading according to treatment groups.

Group	lateral femur	medial femur	lateral tibia	medial tibia	total score
	Median (IQR)				
Channa striatus	1.0 (0.0)^*∗*,*∗∗*^	1.0 (1.0)^*∗*^	1.0 (0.0)^*∗*,*∗∗*^	1.0 (1.0)^*∗*^	4.00 (2.0)^*∗*,*∗∗*^
Glucosamine	3.0 (2.0)	2.0 (1.0)^a^	2.0 (0.0)	2.0 (2.0)	9.00 (3.0)
Control	3.0 (1.0)	3.0 (1.0)	2.0 (1.0)	2.0 (1.0)	10.00 (2.0)

IQR- Interquartile range

^*∗*^ p<0.05 compared with control group

^*∗∗*^ p<0.05 compared with glucosamine group

## Data Availability

The datasets generated and/or analysed of the study are not publicly available but are available from the corresponding author on reasonable request.

## Abbreviations

ACLT: Anterior cruciate ligament transection
Channa: Channa striatus
COMP: Cartilage oligomeric matric protein
COX-2: Cyclooxygenase 2
GAG: Glycosaminoglycan
OA: Osteoarthritis
PGE_2_: Prostaglandin E_2_.
